# The Mini-Chromosome Maintenance (Mcm) Complexes Interact with DNA Polymerase α-Primase and Stimulate Its Ability to Synthesize RNA Primers

**DOI:** 10.1371/journal.pone.0072408

**Published:** 2013-08-20

**Authors:** Zhiying You, Mariarosaria De Falco, Katsuhiko Kamada, Francesca M. Pisani, Hisao Masai

**Affiliations:** 1 Genome Dynamics Project, Department of Genome Medicine, Tokyo Metropolitan Institute of Medical Science, Tokyo, Japan; 2 CNR, Istituto di Biochimica delle Proteine, Consiglio Nazionale Ricerche, Napoli, Italy; 3 Chromosome Dynamics Laboratory, RIKEN Discovery Research Institute, Wako, Saitama, Japan; St. Georges University of London, United Kingdom

## Abstract

The Mini-chromosome maintenance (Mcm) proteins are essential as central components for the DNA unwinding machinery during eukaryotic DNA replication. DNA primase activity is required at the DNA replication fork to synthesize short RNA primers for DNA chain elongation on the lagging strand. Although direct physical and functional interactions between helicase and primase have been known in many prokaryotic and viral systems, potential interactions between helicase and primase have not been explored in eukaryotes. Using purified Mcm and DNA primase complexes, a direct physical interaction is detected in pull-down assays between the Mcm2∼7 complex and the hetero-dimeric DNA primase composed of the p48 and p58 subunits. The Mcm4/6/7 complex co-sediments with the primase and the DNA polymerase α-primase complex in glycerol gradient centrifugation and forms a Mcm4/6/7-primase-DNA ternary complex in gel-shift assays. Both the Mcm4/6/7 and Mcm2∼7 complexes stimulate RNA primer synthesis by DNA primase *in vitro*. However, primase inhibits the Mcm4/6/7 helicase activity and this inhibition is abolished by the addition of competitor DNA. In contrast, the ATP hydrolysis activity of Mcm4/6/7 complex is not affected by primase. Mcm and primase proteins mutually stimulate their DNA-binding activities. Our findings indicate that a direct physical interaction between primase and Mcm proteins may facilitate priming reaction by the former protein, suggesting that efficient DNA synthesis through helicase-primase interactions may be conserved in eukaryotic chromosomes.

## Introduction

In eukaryotic cells, the initiation of DNA replication proceeds in two steps: pre-replication complex (pre-RC) formation and its activation that converts pre-RC into an active replication fork complex. At each step, the Mcm proteins play important roles as a core component of the protein platform for replication initiation and as a key component of the helicase complex which unwinds parental DNA for duplication of leading and lagging strands [1]. *In vitro*, DNA helicase activity was found to be associated with the Mcm4/6/7 complex but not with the Mcm2/3/4/5/6/7 (Mcm2∼7) complex [2], [3], [4], [5], although a recent report show that Mcm2∼7 complex shows helicase activity under specific reaction conditions [6]. Previous works have shown that the Mcm2∼7 complex associates with many other factors during the process of replication. It has been reported that the complex of Mcm2∼7, Cdc45 and GINS (CMG complex) shows an efficient DNA helicase activity [7], [8], leading to the suggestion that CMG is a functional form of the helicase machinery for eukaryotic DNA replication. Furthermore, Mcm10, Ctf4 (And-1), DNA polymerase **ε**, DNA polymerase α-primase, Tof1-Csm3 (Tim-Tipin) and Mrc1 (Claspin), in addition to CMG, were found to generate a multi-molecular assembly in budding yeast [9], [10]. In eukaryotes, the GINS complex, composed of four subunits, Sld5, Psf1, Psf2 and Psf3, is essential for DNA replication and has been implicated at the replication fork. In Archaea, the GINS complex is made of two subunits, interacts with Mcm and primase proteins [11], [12], and is required for chromosomal DNA replication. An archaeal GINS complex stimulates the Mcm helicase activity [12]. Human GINS complex binds to DNA polymerase α-primase and stimulates its DNA synthetic activity [13].

Mammalian DNA polymerase α-primase contains four subunits, p180, p68, p58, and p48 [14], [15]. The DNA polymerase activity resides in the p180 subunit, while the DNA primase activity requires the p58 and p48 subunits that are normally tightly associated with DNA polymerase α. DNA primase catalyzes the synthesis of short RNA oligomers used as primers for DNA synthesis. Primase nonspecifically binds to single-stranded DNA (ssDNA) [16]. Functional cooperation between DNA helicase and primase has been well studied in prokaryotic and viral systems. For examples, in *Escherichia coli*, the replicative DNA helicase DnaB stimulates the DnaG primase action on a naked single-stranded DNA [17], [18], [19], while the primase was shown to stimulate the DnaB helicase activity [20], [21]. The proposed architecture of the replication fork has provided insight into how primase (DnaG)-helicase (DnaB) may interact with each other to facilitate their actions [20], [22], [23]. The model estimates that three molecules of primase bind to one DnaB hexamer. The primase may stabilize the conformation of the DnaB hexamer on DNA, resulting in more processive unwinding. On the other hand, DnaB may facilitate the recognition of target sites for DnaG primase action through its single-stranded DNA binding activity [20], [22], [23].

In T7 phage, a single protein (gp4) contains both primase and helicase activities on separate domains [24], [25], [26], [27], which are related to bacterial DnaG and DnaB, respectively. At the replication fork, priming loop may be generated on the lagging strand through physical association between primase and helicase, resulting in more efficient DNA synthesis through coupling of DNA chain elongation and unwinding and easy handoff to the polymerase [24], [26], [27]. The bacteriophage T4 primase (gp61) also binds tightly to the hexameric T4 helicase (gp41) to form a primosome complex, resulting in increased DNA priming activity [28], [29], [30].

During SV40 replication, T antigen (Tag) physically binds to the DNA polymerase α-primase complex and stimulates its DNA primase and polymerase activities [31], [32], [33]. Mouse DNA helicase B stimulates DNA primase-catalyzed RNA synthesis probably through direct interaction [34]. In addition, the Mcm complex stimulates RNA synthesis by the viral RNA polymerase complex [35]. A protein containing homology to both eukaryotic DNA primase and Mcm was identified on a bacteriophage genome of the bacterium *Bacillus cereus* [36] and displayed not only helicase but also DNA primase and polymerase activities [37]. The presence of both helicase and primase motifs on one single polypeptide is reminiscent of the T7 phage primase-helicase protein. Most recently it was reported that MCM interacts with primase in archaea, and forms ternary complex on DNA [38]. Thus, it would be likely that the mammalian Mcm hexameric helicase also interacts with DNA polymerase α-primase for coordinating unwinding and DNA chain elongation at the replication fork. Human Mcm3 was reported to interact with DNA polymerase α holoenzyme [39].

In this report, we have examined the physical and functional interactions of DNA primase with the Mcm complexes. We show that primer RNA synthesis by primase is stimulated by its interaction with the Mcm complexes.

## Results

### Physical Interactions between Mcm and Primase

**Figure 1:**
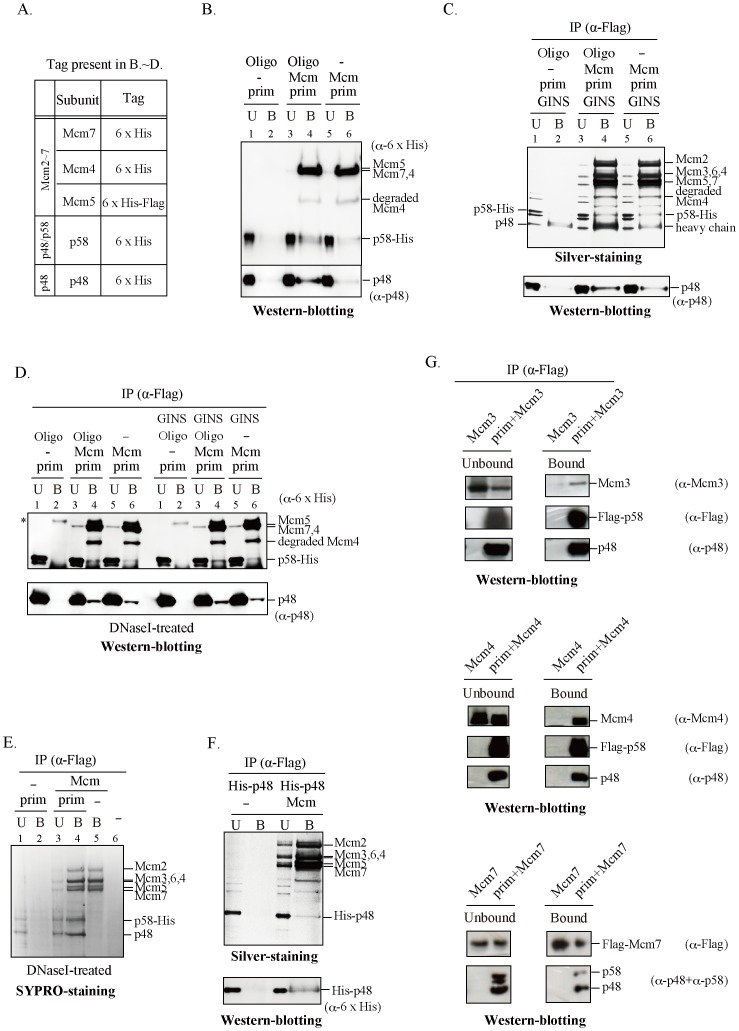
Direct interaction between Mcm assemblies/subunits and primase. (A) The list of the tag present in the Mcm2∼7 complex and p48/p58 or p48 protein used in (B), (C), (D), (E), and (F). The purified Mcm2∼7 complex (1 µg) was mixed with the His-tagged p48/p58 complex (0.25 µg) (B, D, and E), primase (0.25 µg) plus GINS proteins (1 µg) (C and D), or His-p48 (0.5 µg) (F), and immuno-precipitation was performed using anti-Flag M2 antibody beads (Sigma; Flag tag on Mcm5). The bound proteins were eluted with 0.1 M glycine (pH 2.8) (B, C, E, and F) or boiled with SDS sample buffer (D). The eluted samples (shown as “B”) and 1/10 (for silver-staining) or 1/20 (for Western blotting) of unbound fractions (shown as “U”) were analyzed as indicated. The heavy chain is visible in silver staining due to dissociation of the antibody from the beads in first elution (C). DNase I treatment was conducted in the samples without oligonucleotide to remove DNA that might be present in the purified protein fractions. (G) Extracts of Sf9 insect cells expressing the subunits of human DNA primase (p48 and p58) and the indicated Mcm protein were subjected to immuno-precipitation analyses using anti-Flag agarose beads (GE Healthcare). The Flag tag was on p58 in the two upper panels and on Mcm7 in the lower panel. Elution of bound proteins from the beads was carried out using a buffer containing the Flag peptide. For each sample, aliquots of the unbound and bound materials were analyzed by immuno-blotting using the indicated antibodies. Samples were run on 7.5% (B) or 4–20% gradient gel (C, D, E, and F). Star marker * in D indicated a non-special band.

The progression of replication fork requires the interaction of many proteins including that of six Mcm subunits to form a hetero-hexameric complex [1]. In this report, we have explored the possibility that the Mcm helicase and the DNA primase physically and functionally interact with each other at the fork. Immuno-precipitation experiments were conducted to investigate the interaction between the Mcm2∼7 complex and DNA primase or GINS in the absence or presence of an oligonucleotide. Mouse Mcm2∼7 was purified from insect cells infected with recombinant baculoviruses (Fig. S1). Purified Mcm2∼7 (containing the Flag-tagged Mcm5 protein) and primase p48/p58 complexes were mixed with or without an oligonucleotide, and immunoprecipitation using anti-Flag antibody agarose beads were performed (Fig. 1A). p48 and p58 proteins were pulled down by the beads together with each Mcm subunit, as detected by silver staining and Western-blotting, indicating that the Mcm helicase directly interacts with the primase complex. The amount of co-immuno-precipitated p48/p58 slightly increased in the presence of DNA, suggesting that the interaction may be stabilized when DNA is present in the mixture (Fig. 1B, C and D). However, the presence of DNA is not essential for association of Mcm and primase because the interaction of Mcm and primase were also detected in the absence of oligonucleotide and after DNase I treatment (Fig. 1E). Since interaction of GINS with primase and Mcm was reported in Archaea [11], [12], we examined whether the human GINS affects the interaction between Mcm and primase. GINS complex could be pulled down with the anti-Flag antibody beads from the mixture of Mcm2∼7 and GINS proteins (Fig. S2A). However, the interaction between Mcm2∼7 and p48/p58 was not significantly affected by the presence of GINS (Fig. 1D). In addition, we found that both p48/p58 and p48 single subunit binds to Mcm2∼7 in the absence of GINS and oligonucleotide (Fig. 1E and 1F). These data suggest that Mcm binds to primase via the p48 subunit, although interaction with p58 and other subunits of DNA polymerase α-primase cannot be excluded. The data also indicate that this interaction may be stabilized by DNA.

To identify the subunits of Mcm interacting with primase, the human p48/p58 hetero-dimeric complex and a single Mcm subunit was co-expressed in insect cells and co-immuno-precipitation experiments were performed using the anti-Flag antibody agarose beads. The bound proteins were eluted from the resin by the Flag peptide. Mcm3, Mcm4 and Mcm7 but not Mcm2, 5, or 6 were co-immuno-precipitated with the human DNA primase (Fig. 1G and Fig. S2B). Taken together, these results suggest that Mcm directly interacts with primase.

### Mouse Mcm Forms a Complex with the Human Primase or Mouse DNA Polymerase α-primase

**Figure 2:**
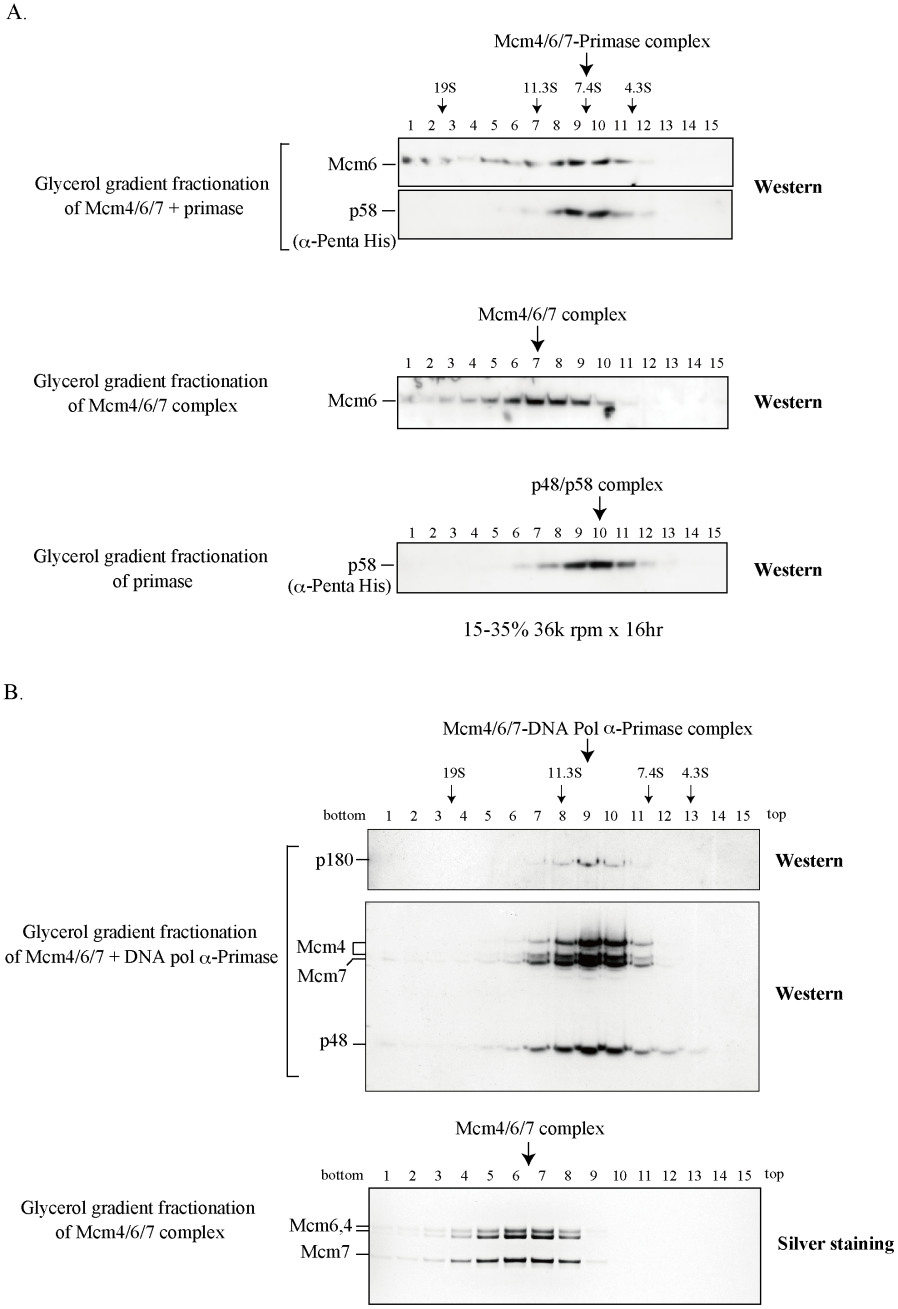
Complex formation of Mcm assemblies with primase or DNA polymerase α-primase. (A) Purified Mcm4/6/7 or primase complex, alone (middle or bottom panel, respectively) or two proteins in combination (upper two panels), were fractionated by centrifugation at 36,000 rpm for 18 h on a 15–35% glycerol gradient in the presence of 1 mM ATP and each fraction was subjected to 4–20% SDS-PAGE, followed by western blotting. The positions of protein markers are indicated. Before loading of protein, the samples were treated by DNase I to prevent DNA influence. Thyroglobulin (19 S), catalase (11.3 S), lactate dehydrogenase (7.4 S), and albumin (4.3 S) (GE Healthcare) were used as protein molecular weight markers. Experiments were carried out twice with similar results and a representative data are shown. (B) Purified DNA polymerase α-primase and Mcm4/6/7 complex were mixed and fractionated by centrifugation at 36,000 rpm for 16 h on a 15–30% glycerol gradient. Protein in each fraction was analyzed by 10% SDS-PAGE and immuno-blotting using the indicated antibodies (upper and middle). Purified Mcm4/6/7 complex alone was fractionated under the same condition and each fraction was analyzed on 7.5% SDS-PAGE, followed by silver staining (lower). The arrows indicate the positions of Mcm4/6/7-primase complex and Mcm4/6/7-DNA polymerase α-primase complex, respectively.

In order to further characterize the interaction between Mcm and primase, we investigated whether the Mcm4/6/7 hexamer could form a complex with primase (p48/p58). Physical interaction between the purified mouse Mcm4/6/7 and human p48/p58 protein complexes was examined by glycerol gradient centrifugation. The purified p48/p58 protein sedimented predominantly in the fractions 10. Extrapolating from the standards, the sedimentation coefficient of this complex is 5.9 S (Fig. 2A, lower), whereas the Mcm4/6/7 complex sedimented at around fractions 7 (Fig. 2A, middle). Our previous data indicated that the Mcm4, Mcm6, and Mcm7 proteins co-sedimented and peaked around the protein marker catalase in glycerol gradient centrifugation. However, in a native gel, a band of 550 kDa was detected, suggesting that the Mcm4/6/7 forms a hexameric complex (a dimer of two trimers) (Fig. S1) [3], [5]. When p48/p58 was mixed with Mcm4/6/7, p58 co-sedimented with the Mcm6 subunit at fractions 9–10 (Fig. 2A, top), suggesting the association of p48/p58 with the Mcm4/6/7 complex. The complex formation between Mcm4/6/7 and primase was examined in native gel analysis as well (Fig. S3A). The molecular mass of the hexameric Mcm4/6/7 alone containing a dimer of the trimer is ∼550 kDa. In the presence of p48/p58, however, a new complex around 232 kDa is detected, which likely contains p48/p58. Thus, primase interacts with the Mcm4/6/7 complex, somehow disrupts the hexameric structure and forms a complex with the Mcm4/6/7 trimer.

We also examined the interaction between the purified mouse Mcm2∼7 and human p48/p58 protein complexes by glycerol gradient centrifugation and the native gel analysis. When the p48/p58 hetero-dimer was mixed with Mcm2∼7, a portion of p48 co-sedimented with the Mcm2∼7 complex in glycerol gradient centrifugation (data not shown). However, the majority of p48/p58 remained at low-molecular-weight position, suggesting that the interaction of Mcm2∼7 with primase is unstable and that only a limited portion of primase formed a complex with Mcm2∼7 under the experimental condition. In native gel analysis, Mcm2∼7 alone predominantly formed a 600-kDa species and the p48/p58 complex migrated as multiple sizes under the condition employed. Addition of the primase complex to the Mcm2∼7 hetero-hexamer resulted in generation of a small amount of a new protein assembly larger than 660 kDa (Fig. S3B). These results indicate that the Mcm2∼7 complex also associates with the hetero-dimeric primase complex.

Potential interaction between the mouse Mcm4/6/7 complex and mouse DNA polymerase α-primase tetrameric complex was also explored by using glycerol gradient sedimentation. The Mcm4 and Mcm7 subunits of Mcm4/6/7 co-sedimented with the p180 and p48 subunits at fraction 9, as shown by immuno-blotting (Fig. 2B, top and middle), suggesting interaction between Mcm4/6/7 and DNA polymerase α-primase. The Mcm4/6/7 complex alone peaked between fraction 6 and 7 in silver staining (Fig. 2B, bottom). DNA polymerase α-primase alone peaked at fraction 10, consistent with the previous report that the mouse DNA polymerase α-primase tetrameric complex sedimented at 9.1 S between the catalase (11.3 S) and alcohol-dehydrogenase (7.4 S) in glycerol gradient centrifugation [40]. This result suggests a possibility that Mcm4/6/7 forms a complex with DNA polymerase α-primase. The size of the complex suggests the presence of a single trimer of Mcm4/6/7 and a single polymerase α-primase in the peak fraction.

### Influence on the DNA-binding Activities of Mcm4/6/7 and Primase

**Figure 3:**
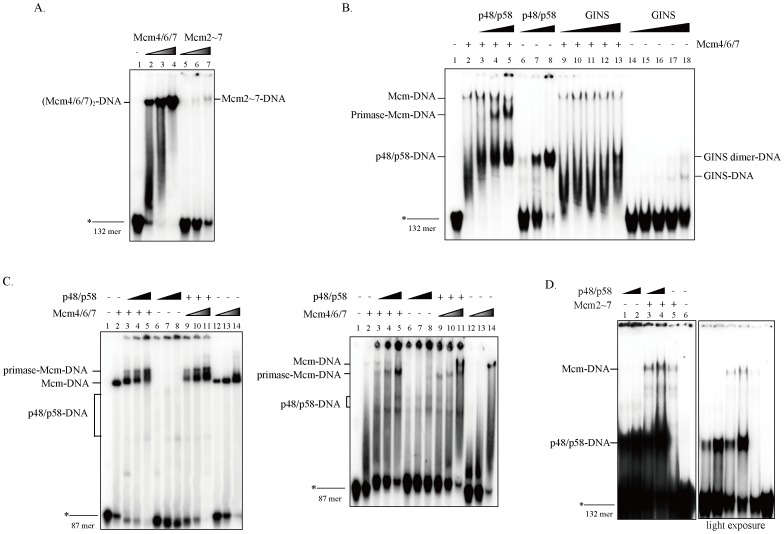
Effect of primase on DNA-binding activity of Mcm4/6/7 and Mcm2∼7. DNA-binding activities on ssDNA were examined in gel shift assays. (A) The proteins added were; 37.5 ng (lane 2), 75 ng (lane 3) and 150 ng (lane 4) of Mcm4/6/7; 75 ng (lane 5), 150 ng (lane 6), and 300 ng (lane 7) of Mcm2∼7. A constant amount of Mcm4/6/7 (15 ng/2.3 nM) in (B) or Mcm2∼7 (360 ng/50 nM) in (D) and various amounts of the p48/p58 primase and GINS were added. The proteins added were: 30, 60, and 120 ng of primase (25 nM, 50 nM and 100 nM, respectively, as a monomer) and 30, 60, 120, 250, and 500 ng of GINS (25 nM, 50 nM, 100 nM, 200 nM and 400 nM, respectively) in B, and 60 and 120 ng of primase in D. The reaction mixtures were incubated with 20 fmole labeled substrate (132 mer) at 30°C for 30 min, and were applied on a 5% native polyacrylamide gel (32.3:1) containing 1x TBE, followed by detection with autoradiography. In (C), a constant amount of Mcm4/6/7 (50 ng) or various amounts of Mcm4/6/7 (25 ng, 50 ng and 100 ng), and a constant amount of p48/p58 (50 ng) or various amounts of the p48/p58 primase (25 ng, 50 ng and 75 ng) were added. The DNA-binding reactions were separated, and two-thirds of reaction mixtures were incubated with oligonucleotide DNA (37mer-dT_50_), and then the reactions were separated. A half was run in 5% native gel (acrylamide: bis = 37.5:1) containing 5% glycerol, 0.5x TBE (left), and the rest was run in 5% native gel (acrylamide: bis = 32.3:1) containing 1x TBE (right).

It has been known that Mcm4/6/7 binds to single-stranded DNA with a high affinity, but hardly binds to double-stranded DNA [41]. In contrast, the Mcm2∼7 complex exhibited very weak single-stranded DNA binding in gel-shift assay, while it showed single-stranded DNA binding comparable to that by Mcm4/6/7 in filter binding assays [42]. This could be due to unstable association of Mcm2∼7 with ssDNA. We examined DNA binding activities of the purified mouse Mcm4/6/7 and Mcm2∼7. Consistent with previous studies, the Mcm2∼7 complex showed very weak binding to ssDNA in the presence of the ATP-γ-S in mobility shift assays compared to Mcm4/6/7 complex (Fig. 3A) [42]. We next examined DNA-binding activities of the mouse Mcm and human p48/p58 complex using a 132-mer pyrimidine-rich oligonucleotide DNA that is known to be tightly bound by eukaryotic primase [43]. Strong single-stranded DNA-binding activity was detected with primase, and Mcm4/6/7 hexamer also binds to ssDNA (Fig. 3B). In the presence of both primase and Mcm4/6/7, a band migrating slower than Mcm4/6/7 alone was detected and its intensity was proportional to the increasing concentrations of the primase (Fig. 3B, lanes 3–5), suggesting the formation of a complex containing both Mcm4/6/7 and primase on single-stranded DNA. We speculate that the hexameric Mcm4/6/7 [(Mcm4/6/7)_2_] was destructed and converted to a complex containing a single Mcm4/6/7 trimer and p48/p58 proteins under the condition of the study. To confirm this possibility, the same reactions were separated under different gel electrophoresis conditions (Fig. 3C). Under the gel electrophoresis condition same as Figure 3B, (Mcm4/6/7)_2_-DNA shifted to a faster-migrating band upon addition of primase, which is presumably Mcm4/6/7(trimer)-primase complex (Fig. 3C, right panel). These are similar to the observation in Figure 3B. Under another gel electrophoresis condition using a gel containing 5% glycerol, a slower-migrating form appeared upon addition of primase or Mcm4/6/7, which may represent a (Mcm4/6/7)_2_-primase-DNA ternary complex (Fig. 3C, left panel). The intensities of ternary complexes significantly increased in response to increased protein amount of p48/p58 (lanes 3–5) or Mcm4/6/7 proteins (lanes 9–11), in the presence of a constant amount of Mcm4/6/7 or primase, respectively (Fig. 3C, left panel and Fig. S4A). These results strongly support the presence of Mcm4/6/7-primase-DNA complex. However, they also suggest that the complex of Mcm4/6/7 and primase is not stable under some gel electrophoresis condition and the Mcm4/6/7 hexamer may be disrupted. The intensities of primase-induced shifted bands on a 87mer and a 132mer appear quite different under the same reactions (compare Fig. 3C and Fig. S4B). This is probably due to the difference of sequence compositions of the two oligonucleotides. In contrast, GINS alone interacts with single-stranded DNA only weakly (Fig. 3B, lanes 14–18). Although GINS interacts with Mcm (Fig. S2A), the migration of Mcm4/6/7-DNA did not change by the addition of increased amount of GINS (Fig. 3B, lanes 9–13), suggesting that, unlike primase, human GINS could not form a complex with Mcm4/6/7 on DNA.

Mcm2∼7 generates very little complex on ssDNA in gel shift assays (Fig. 3A). In the presence of the primase, slow-migrating bands appeared, which may represent Mcm2∼7-DNA complex or Mcm2∼7-primase-DNA ternary complex (Fig. 3D). The complex may not contain primase and primase may simply stimulate the complex formation between Mcm and DNA, since the complex formation between primase and Mcm2∼7 is weak (Fig. S3B) and the Mcm2∼7-primase-DNA ternary complex may be unstable and disassemble during the migration in the gel. Taken together, these results suggest that the Mcm complexes and primase interact on DNA and that the primase increases the association of the Mcm complexes with DNA.

### Effect of Primase on Mcm Helicase and ATPase Activities

**Figure 4:**
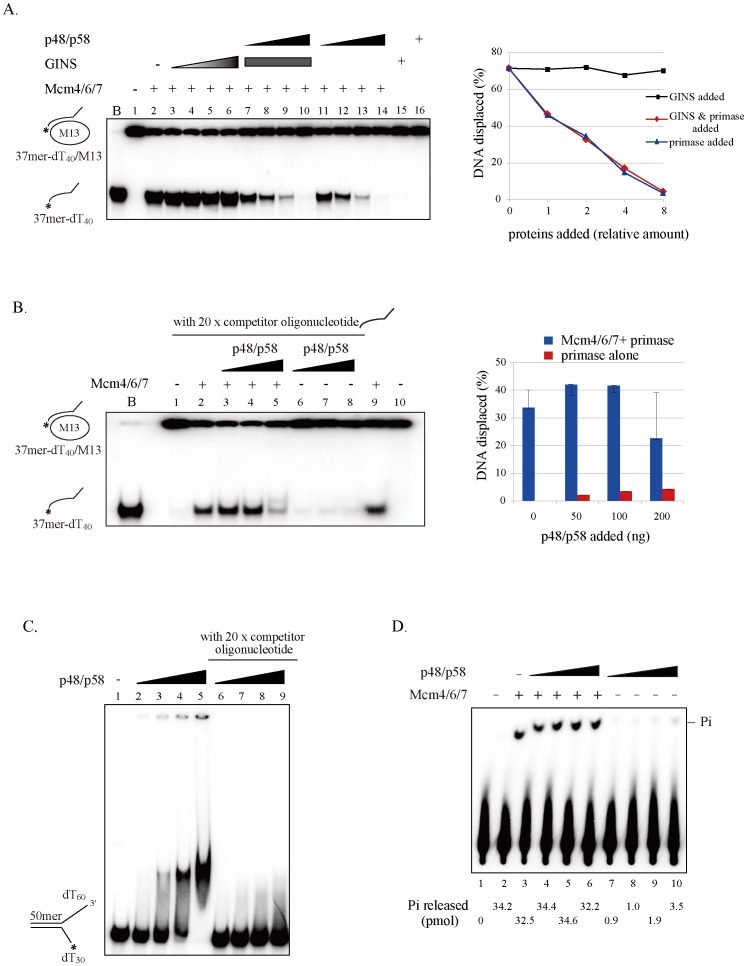
Effect of primase on DNA helicase and ATP hydrolysis activities of Mcm4/6/7. (A and B) DNA helicase activities were examined with a constant amount of Mcm4/6/7 (50 ng/7.5 nM (A) or 40 ng/6 nM (B)) and various amounts of primase or GINS using a partial hetero-duplex substrate (10 fmoles). The proteins added in (A) were; 25 ng, 50 ng, 100 ng and 200 ng of p48/p58 primase, and 125 ng, 250 ng, 500 ng and 1 µg of GINS, and constant amount of GINS (500 ng) in lanes 7–10. In (B), 50 ng, 100 ng, and 200 ng of the p48/p58 primase were added. In lanes 1 to 8, 20-fold excess of cold competitor oligonucleotide DNA (37mer-dT_40_) was also added. The samples were incubated at 37°C for 1 hr and were then subjected to electrophoresis through a 10% polyacrylamide gel in 1x TBE buffer. B, boiled substrate DNA. The displaced oligonucleotide in (A) and (B) were quantified and showed as a graph in right side. Error bars in (B) represent the standard deviation from at least three independent experiments. (C) In the presence of competitor oligonucleotide, DNA-binding activities were examined with various amounts of primase protein (12.5 ng, 25 ng, 50 ng and 100 ng) on the fork substrate (25 fmoles). The reaction mixture was incubated at 30°C for 30 min and were run a 5% nondenaturing polyacrylamide gel. 20-fold unlabeled oligonucleotide DNA (50mer-dT_30_) was added as competitor from lanes 6 to 9. (D) ATPase activity of a constant amount of Mcm4/6/7 (150 ng) was measured in the presence of increasing amounts of p48/p58 primase (62.5 ng, 150 ng, 300 ng and 600 ng) and a 87 mer oligonucleotide (20 fmole). The amount of released phosphate (Pi, pmoles) was quantitated and labeled at the bottom of the autoradiogram.

To examine whether primase could affect the helicase activity of the Mcm complex, as demonstrated for DnaG primase and DnaB helicase in *E. coli* [20], [21]. DNA helicase and ATPase activities of the Mcm4/6/7 were assayed in the presence of the p48/p58 primase. Mcm complex (7.5 nM, Mcm4/6/7 hexamer) was tested for unwinding in the absence and presence of 20 nM∼160 nM p48/p58 hetero-dimer with a partial duplex substrate (10 fmole). The Mcm4/6/7 helicase activity was inhibited by the addition of increasing amounts of primase protein (Fig. 4A, lanes 11–14). This inhibition is likely to have occurred due to substrate competition between Mcm and primase. In fact, addition of a 20-fold excess cold competitor oligonucleotide resulted in restoration of DNA unwinding activity by Mcm4/6/7 in the presence of the primase complex (Fig. 4B, lanes 3–5). Indeed, the DNA-binding activity of primase was inhibited in the presence of 20-fold excess cold competitor oligonucleotide (Fig. 4C, compare lanes 2–5 to lanes 6–9). Even under this condition, primase did not significantly stimulate the helicase action of Mcm (Fig. 4B). The doublet bands seen in lane 5 of Fig. 4B, could be due to the binding of primase to the displaced oligonucleotide. Indeed, extensive pre-treatment eliminated the doublet (Fig. S5, compare lanes 3–5 with lanes 9–11). The inhibition of Mcm4/6/7 helicase activity by primase could also be caused by the conversion of the helicase-active hexameric Mcm4/6/7 complex to a helicase-inactive trimeric Mcm4/6/7-primase complex. It has been reported that archaeal GINS stimulates Mcm helicase activity [12]. However, the human GINS complex did not display any effect either on the helicase activity of the Mcm4/6/7 complex (Fig. 4A, lanes 3–6) or on the inhibition of Mcm4/6/7 helicase by primase (Fig. 4A, compare lanes 7–10 to lanes 11–14).

DNA helicase activity depends on a set of sub-activities, including DNA binding and ATP hydrolysis and coordination of these activities is required to efficiently unwind DNA [44]. The primase (p48/p58 complex) alone did not show any ATPase activity in the presence of single-stranded DNA (Fig. 4D). Addition of primase (p48/p58 complex) did not increase the ATPase activity of Mcm, consistent with the absence of effect on the Mcm DNA helicase function.

### The Mcm Complex Stimulate RNA Primer Synthesis by Primase

**Figure 5:**
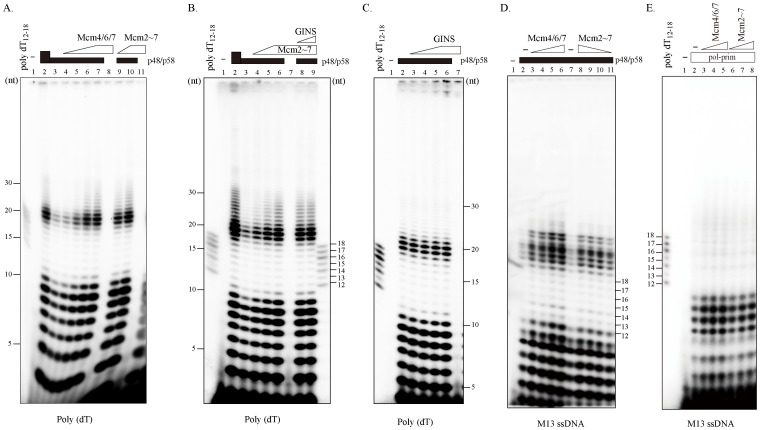
Stimulation of RNA primer synthesis by Mcm complexes. RNA primer was synthesized by p48/p58 primase in the absence or presence of Mcm and/or GINS complex on poly(dT) (A-C) or on M13 mp18 ssDNA (D). RNA primer synthesis by DNA polymerase α-primase on M13 mp18 ssDNA substrate was examined in the absence or presence of Mcm complex (E). After incubation for 1 hr at 37°C, the products were applied on 20% denaturing polyacrylamide gel in 1x TBE buffer. A labeled oligonucleotide (dT_12–18_) and 10-bp DNA ladder were used as a size marker. (A) The proteins added were; 100 ng (lanes 3–7, 9 and 10) and 300 ng (lane 2) of p48/58 primase; 100 ng (lane 4), 200 ng (lane 5), 400 ng (lane 6), and 600 ng (lanes 7 and 8) of Mcm4/6/7; or 200 ng (lane 9) and 400 ng (lanes 10 and 11) of Mcm2∼7. (B) The proteins added were; 100 ng (lanes 3–6, 8 and 9) and 300 ng (lane 2) of p48/58 primase; 100 ng (lane 4), 200 ng (lane 5) and 400 ng (lanes 6–9) of Mcm2∼7; and 0.5 µg (lane 8) and 1 µg (lane 9) of GINS. (C) The proteins added were; 100 ng of p48/58 (lanes 2–6) and 0.25 µg (lane 3), 0.5 µg (lane 4), 1 µg (lane 5) and 1.5 µg (lanes 6 and 7) of GINS. (D) The proteins added were; (lanes 2–11) 100 ng of p48/58 primase; 100 ng (lane 3), 200 ng (lane 4), 400 ng (lane 5), and 600 ng (lane 6) of Mcm4/6/7; or 50 ng (lane 11), 100 ng (lane 10), 150 ng (lane 9), and 200 ng (lanes 8) of Mcm2∼7. (E) The proteins added were; (lanes 2–8) 100 ng of human DNA polymerase α-primase complex; 100 ng (lane 3), 200 ng (lane 4), and 300 ng (lane 5) of Mcm4/6/7; or 50 ng (lane 6), 100 ng (lane 7), and 150 ng (lane 8) of Mcm2∼7. More than three independent experiments were carried out.

Next, we examined whether the RNA primer synthesis would be affected by Mcm4/6/7 or Mcm2∼7 complexes. We first observed that the RNA synthesis increased in proportion to the primase added using the poly(dT) as template (Fig. 5A and 5B, lanes 2–3). In contrast, the Mcm4/6/7, Mcm2∼7 and GINS complexes by themselves did not show any RNA primer synthesis activity. Next, we examined the effect of addition of Mcm4/6/7 or Mcm2∼7 on the primase synthesis function (Fig. 5A, lanes 4–7 or lanes 9–10, and 5B, lanes 4–6). Both Mcm4/6/7 and Mcm2∼7 stimulated primer synthesis by approximately three-fold. In contrast, GINS did not affect the primase activity (Fig. 5C, lanes 3–6). RNA synthesis by primase in the presence of Mcm was also not affected by GINS (Fig. 5B, lanes 8–9). Under optimal reaction conditions that utilize M13 ssDNA template, the priming ability of primase significantly increased in the presence of both the Mcm4/6/7 and Mcm2∼7 complex (approximately 4–7 folds and 2.3–3.3 folds, respectively; Fig. 5D, lanes 3–6 and lanes 8–11), consistent with above result. In addition, the Mcm complexes stimulated the primase activity of the hetero-tetrameric DNA polymerase α-primase complex as well (Fig. 5E). However, Mcm did not have any effect on DNA synthesis on a singly-primed M13mp18 single-stranded DNA template by DNA polymerase α-primase (data not shown).

### Mutual Stimulation of Mcm and Primase in Single-stranded DNA-binding

**Figure 6:**
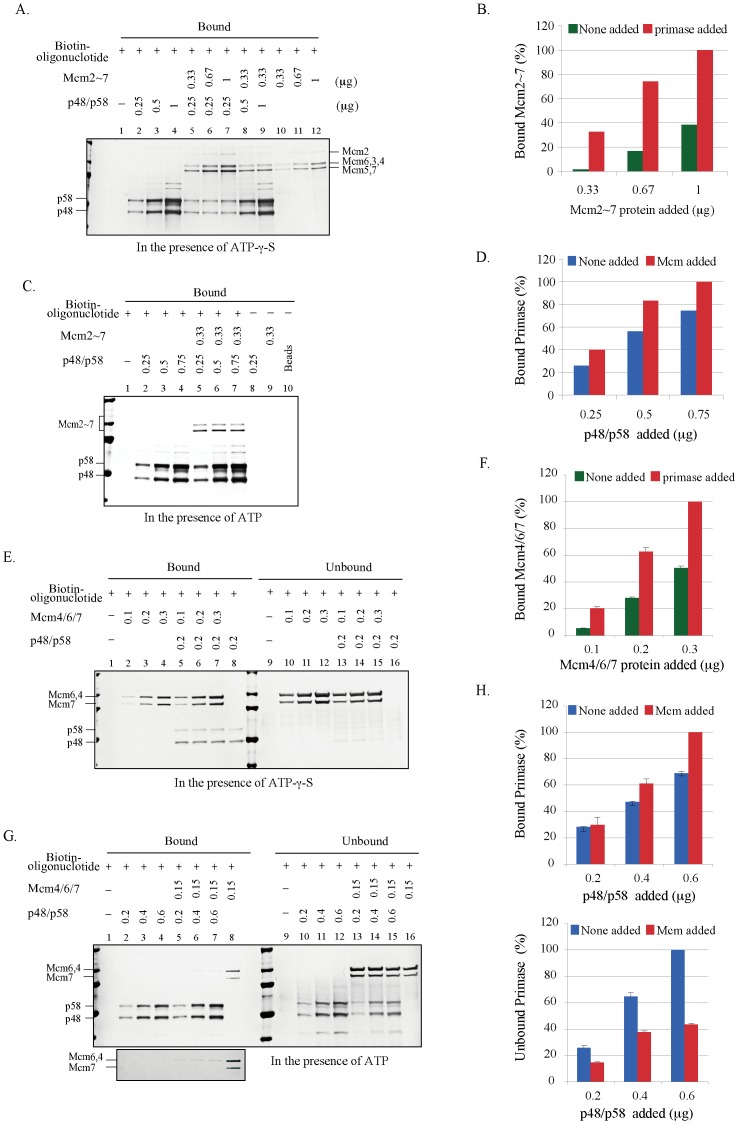
Single-stranded DNA binding activities of primase and Mcm2∼7 proteins. (A) and (C) the p48/p58 primase and/or Mcm2∼7 complex as shown in the figure were mixed with biotin-labeled 37mer-dT_40_ oligonucleotide (50 pmole) in the presence of 0.5 mM ATP-γ-S (A) or 1 mM ATP (C). (E) and (G) the p48/p58 primase and/or Mcm4/6/7 complex were mixed with biotin-labeled 37mer-dT_40_ oligonucleotide (50 pmole) in the presence of 0.5 mM ATP-γ-S (E) or 1 mM ATP (G). After incubation at 30°C for 30 min, the streptavidin-coated magnetic beads were added. Then, the beads were washed, re-suspended in SDS sample buffer and analyzed on 4–20% SDS-PAGE, followed by silver staining. (B) The amounts of Mcm2∼7 subunits in lanes 5–7 and 10–12 in (A) were quantified, and the levels relative to the maximum level of Mcm (lane 7; taken as 100) are presented. (D) The amounts of primase subunits in lanes 2–4 and 5–7 in (C) were quantified, and the levels relative to the maximum level of primase in the presence of Mcm (lane 7; taken as 100) are presented. (F) The amounts of Mcm4/6/7 subunits in lanes 2–4 and 5–7 in (E) were quantified, and the levels relative to the maximum level of Mcm in the presence of primase (lane 7; taken as 100) are presented. (H) The amounts of primase subunits in lanes 2–4 and 5–7 in (G) were quantified, and the levels relative to the maximum level of primase in the presence of Mcm (lane 7; taken as 100) are presented (upper). The amounts of primase subunits in lanes 10–12 and 13–15 in (G) were quantified, and the levels relative to the maximum level of primase in the absence of Mcm (lane 12; taken as 100) are presented (lower). Error bars represent the standard deviation from at least three independent experiments. A dark figure (a section of Mcm4/6/7 in Figure 6G) is shown in the bottom.

To examine the loading of primase and helicase onto DNA, pull-down assays of primase and Mcm2∼7 were carried out by using a biotin-labeled oligonucleotide in the presence of ATP-γ-S, an analog of ATP, which is strongly bound but poorly hydrolyzed by the Mcm complex. We show that the primase protein alone efficiently binds to the oligonucleotide, indicating that the affinity of primase to single-stranded DNA is very strong (Fig. 6A, lanes 2–4), consistent with the result of gel shift assays (Fig. 3B). In contrast, the bulk of Mcm2∼7 is present in the unbound fractions (Fig. S6), indicating that the affinity of Mcm2∼7 to single-stranded DNA is low (Fig. 6A, lanes 10–12), consistent with the results of gel shift assays (Fig. 3A and 3D). Under this condition, the DNA-binding activity of the Mcm2∼7 complex significantly increased in the presence of primase (Fig. 6A, compare lanes 5–7 to lanes 10–12 and Fig. 6B), suggesting that primase facilitates binding of Mcm to single-stranded DNA. This is consistent with the result of gel shift assay (Fig. 3D). It was proposed that the ATP-DnaB-single-stranded DNA complex provides a recognition signal for primase, thereby promoting priming [45]. In a subsequent set of DNA-binding assays, ATP-γ-S was substituted with ATP and the oligonucleotide was added to the reaction mixtures last. Under this condition, a small increase of primase binding to DNA was observed in the presence of Mcm2∼7 (Fig. 6C, compare lanes 2–4 to lanes 5–7, and Fig. 6D). Similar results were also observed between the Mcm4/6/7 and primase (Fig. 6E and 6G). In the presence of a constant amount of primase, the DNA-binding activity of Mcm4/6/7 obviously increased (Fig. 6E, compare lanes 2–4 to lanes 5–7 and Fig. 6F), while the unbound Mcm4/6/7 decreased in proportion to the amount of DNA-bound primase (Fig. 6E, compare lanes 10–12 to lanes 13–15). On the other hand, the DNA binding ability of primase increased in the presence of Mcm4/6/7 (Fig. 6G, compare lanes 2–4 to lanes 5–7 and Fig. 6H, upper), and the DNA-unbound primase decreased in the presence of Mcm4/6/7 (Fig. 6G, compare lanes 10–12 to lanes 13–15 and Fig. 6H, lower). We noticed that the increase of primase in the bound fraction in the presence of Mcm4/6/7 does not precisely reflect its decrease in the unbound fraction (compare Fig. 6H, lower to upper). This could be due to the instability of primase-DNA-Mcm complex during the wash process of the bound beads. The DNA-binding of Mcm4/6/7 decreases in the presence of primase and ATP (Fig. 6G, compare lanes 5–7 to lane 8). Two possibilities are conceivable. Primase may disrupt the helicase-active hexameric Mcm4/6/7 complex and convert it to a trimeric Mcm4/6/7 complex that is deficient in the DNA binding activity as well as in DNA helicase activity. This may also partly explain inhibition of Mcm4/6/7 helicase activity by primase (Fig. 4A). Alternatively, ATP hydrolysis of Mcm4/6/7 may cause the conformational change in Mcm4/6/7 and cause it to move on single-stranded DNA. This may be associated with increased interaction of primase with DNA, which may results in decreased DNA-binding activity of Mcm4/6/7. We have concluded that the interaction between primase and Mcm may lead to mutual stimulation of their binding to single-stranded DNA.

## Discussion

Physical and functional interaction between the replicative helicase and primase has been well known in bacteria and bacteriophages. We show here for the first time that the Mcm helicase forms a complex with DNA polymerase α-primase through direct interaction with the primase subunits. We also show that the primase activity is stimulated by the Mcm complexes. In the prokaryotic and animal viral replisomes, physical links between DNA helicase, DNA polymerase and primase regulate fork progression and allow unwinding to be coordinated with DNA chain elongation [14], [15], [24]. Our findings suggest a possibility that similar mechanisms may operate for eukaryotic replisomes.

It was proposed that DnaB helicase activates DnaG primase by serving as a docking station to increase the local concentration of single-stranded DNA template relative to primase [22]. We have shown that Mcm physically interacts with primase probably through interaction between p48 and Mcm3/Mcm4/Mcm7 subunits (Fig. 1 and 2). Mcm and primase form a complex on DNA and mutually stimulate their ssDNA-binding activities, suggesting that they cooperate with each other to facilitate fork progression and DNA chain elongation. Indeed, RNA primer synthesis is stimulated significantly by both the helicase-active Mcm4/6/7 and Mcm2∼7 complexes (Fig. 5). Thus, interactions between Mcm and primase may be important for stimulation of the priming activity. This is similar to the previous report that a helicase-dead DnaB can stimulate DnaG primase [46].

Unlike in *E. coli* and T4, in which primases stimulate helicase activity of replicative helicases, primase inhibits the Mcm4/6/7 helicase activity (Fig. 4). We speculate that primase competes out Mcm4/6/7 on DNA substrate due to its strong single-stranded DNA binding activity, since the inhibition is abolished by addition of excess competitor DNA. An alternative possibility is that primase disrupts helicase-active hexameric Mcm4/6/7 complex (Fig. 2A), in a manner similar to the inhibition of the Mcm4/6/7 helicase by Mcm2 or Mcm5/3 complex [47].

Mcm is a 3′ to 5′ helicase present on the leading strand [2], [3], [4], [7], and thus, it would need to interact with the primase acting on the other strand [48]. It has been known that the eukaryotic polymerase and primase are associated with each other in a highly flexible manner [49]. The primase could interact with different surfaces of polymerase, potentially giving rise to a flexible movement in association with the moving polymerase. Thus, it is conceivable that primase present on the lagging strand interacts with the Mcm complex on the leading strand. The purified p48/p58 complex migrates as a multimer in native gel under some condition, although similar levels of intrinsic RNA primer synthesis activity were always observed (our unpublished data). It could be stabilized as a monomeric primase when it forms a complex with DNA polymerase α. In addition, the interaction between Mcm and primase is weak due to the transient nature of their association. Gel-shift experiments under various gel electrophoresis condition suggested that the MCM-primase complex is unstable even with DNA. GINS and Cdc45 interact with both polymerase α-primase and Mcm proteins [11], [50], [51]. Thus, the CMG complex may more efficiently recruit polymerase α-primase to the replication fork and generate more stable complex. We have not found in our *in vitro* assays any stimulatory effect of Mcm on the DNA synthesis catalyzed by the DNA polymerase α-primase complex. In contrast, the SV40 T-antigen helicase interact with all four subunits of the polymerase α-primase complex and can stimulate both primase and DNA chain elongation activities [31], [32], [33]. The failure of Mcm to stimulate DNA synthesis by DNA polymerase α-primase *in vitro* may suggest that other replication fork proteins may be required for full stimulation of DNA chain elongation. In fact, a large replisome progression complex (RPC) containing GINS, Mcm, Cdc45, Mrc1, Tof1-Csm3, FACT, Ctf4/And-1, Mcm10 and DNA topoisomerase I was detected in budding yeast [9], [10], [52]. It was reported that the GINS-Ctf4 complex of the RPC is crucial to couple Mcm2∼7 to DNA polymerase α [10]. Additional factors, such as Mcm10 and Cdc45, are also known to bind to DNA polymerase α at the replication fork [9], [51], [53]. More recently, studies in yeasts indicated a role of Mcm10 in activation of the CMG helicase [54], [55]. Effects of these factors on priming and DNA synthesis activities of DNA polymerase α-primase need to be carefully examined in the future.

Our binding assays show that primase directly interacts with the Mcm3, 7, and 4 subunits (Fig. 1G), which constitute a half surface of the hexameric Mcm ring. On the other hand, GINS and Cdc45 were reported to interact with Mcm3, Mcm5 and Mcm2, an adjacent another surface of the Mcm ring [56]. However, our biochemical studies showed that GINS stimulates neither the interaction between Mcm and primase nor the RNA synthesis by primase in the presence or absence of Mcm (Fig. 1D and 5). Thus, the architecture of eukaryotic replisome may be different from that in archaea where GINS acts as a linker (bridge) between the primase and Mcm helicase [11], [12].

Our results suggest that Mcm forms a specific complex with DNA polymerase α-primase at the replication fork and that this interaction may facilitate RNA primer synthesis on the lagging strand. This probably represents only a portion of many protein-protein interactions, which occur within the replisome complex. We propose that Mcm, moving on the leading strand, serves as a part of the bridge that links DNA polymerase α-primase so that primer synthesis occurs efficiently on the lagging strand. Our results also suggest the conservation of helicase-primase interaction at both prokaryotic and eukaryotic replication forks [22], [24], [27], [30], [57]. Future analyses using the helicase-proficient CMG complex as well as other associated fork factors will provide further insight into the functional and physical interactions that underpin the molecular architecture of the highly efficient and versatile eukaryotic replisome complex.

## Materials and Methods

### Expression and Purification of Recombinant Proteins in Insect Cells and *E. coli*

The highly purified recombinant mouse Mcm4/6/7 and Mcm2∼7 protein complexes were prepared from insect cells as follows. For expression of the Mcm4/6/7, High Five cells were co-infected with recombinant baculoviruses expressing the His_6_-Mcm4/Mcm6 proteins and those expressing the Mcm7-Flag, and were collected at 48 hr post-infection. The recombinant Mcm4/6/7 complex was purified as previously described [41]. For expression of the Mcm2∼7 complex, Sf9 or High Five cells were co-infected with the combination of Mcm2/Mcm7-His_6_, His_6_-Mcm4/Mcm6, and Mcm3/Mcm5-His_6_-Flag baculoviruses, and were collected at 48 hr post-infection. The Mcm2∼7 complex was purified with consecutive steps involving nickel-agarose affinity chromatography, anti-Flag M2 antibody agarose affinity chromatography, and glycerol gradient sedimentation. Sf9 and High Five insect cells were cultured at 27°C in Sf-900 II SFM (Life Technologies, Inc.) and EX-CELL 405 medium (JRH Biosciences), respectively.

His-tagged p58 and non-tagged p48 complex or His-tagged p48 alone was over-produced in the *E. coli* BL21 (DE3) RIL strain and purified as previously described [58].

### Reagents

Labeled and unlabeled dNTPs/rNTPs were purchased from GE Healthcare. M13mp18 single-stranded circular DNA (ssDNA) and T4 polynucleotide kinase (T4 PNK) were obtained from TAKARA. Anti-Flag M2 antibody-agarose beads, anti-Flag M2 antibody, Flag peptide, Adenosine-5′-triphosphate (ATP), and adenosine 5′-(gamma-thio) triphosphate (ATP-γ-S) were purchased from Sigma. Anti-penta-His and DNA pol α (p180) antibodies were purchased form Qiagen and Santa Cruz, respectively. Anti-GINS antibodies were made by laboratory. Oligonucleotides were obtained from Hokkaido System Science Co., Ltd. (Hokkaido, Japan).

### Construction of DNA Substrates

5′-tailed partial hetero-duplex substrates were constructed by annealing a dT_40_-37mer oligonucleotide to M13mp18 ssDNA. The oligonucleotide carrying the 40 mer oligo (dT) tail at the 3′ end of the hybridizing 37 mer sequence was first labeled with [γ-^32^P]ATP and T_4_ PNK, and then annealed to M13mp18 ssDNA. The reaction mixture was heated at 95°C, kept at 67°C for 1 hr, and then allowed to slowly cool down to 30°C. The labeled substrates were purified by Sepharose CL4B column chromatography (GE Healthcare). The 132 mer oligonucleotide (5′-CCACACATGATTTGTTTGCTCCCTGAAATGATCTATATTTAATATATAATGTATATTCCCTCGGGATTTTTTATTTTGTGTTATTCCACGGCATGAAAAACAAAAAACATTCTTCTCATCCTTGGTCCCTCA-3′) and 37mer-dT_50_ (87 mer) [41] were labeled with [γ-^32^P]ATP and T_4_ PNK. The Y-fork substrate composed of a 50 nucleotides duplex region as well as a 30 mer oligo (dT) tail at the 5′ and a 60 mer oligo (dT) tail at the 3′ end [41]. The double-stranded DNA (173 bp) was labeled by filling in the cohesive ends with [α-^32^P]dCTP and the Klenow fragment of DNA polymerase I.

### Primer RNA Synthesis Assays

Primer RNA synthesis was carried out using poly(dT) or M13 ssDNA as templates. The reaction mixtures with poly(dT) (2 nmole as nucleotide) contained 0.1 mM [α-^32^P]ATP (3 μCi/nmol), 20 mM Tris-HCl (pH 7.5), 10 mM magnesium acetate, 5 mM dithiothreitol (DTT), and 0.1 mg/ml bovine serum albumin (BSA). Whereas the reaction mixture with 200 ng of M13mp18 ssDNA (0.6 nmol of nucleotide concentration) contained 4 mM ATP, 200 μM CTP, 200 μM UTP, 2 μM GTP and 5 μCi [α-^32^P]GTP, 20 mM Tris-HCl pH 7.5, 10 mM magnesium actate, 2 mM DTT, 0.25 mg/ml BSA, 40 mM creatine phosphate, 100 μg/ml creatine kinase. After addition of primase or DNA polymerase α-primase and each protein as indicated, assays were incubated for 1 hr at 37°C. The products were ethanol-precipitated in the presence of 2.5 μl of 3 M sodium acetate (pH 5.2) and 10 μg of *E. coli* tRNA, washed with 75% ethanol, dried, re-suspended in a solution containing 80% de-ionized formamide, 5 mM ethylenediaminetetraacetic acid (EDTA) and 0.05% bromphenol blue. Primer RNAs were analyzed on denaturing polyacrylamide gel electrophoresis. Samples were heated at 95°C for 3 min and were loaded onto 20% polyacrylamide gel (acrylamide: bisacrylamide ratio, 19:1) containing 7 M urea and 0.5x Tris-Borate-EDTA (TBE) buffer. As a molecular weight marker, labeled oligo (dT_12–18_), 10-bp DNA ladder, and 50-bp DNA ladder were used.

### DNA-binding and Helicase Assays

DNA helicase and DNA-binding activities were examined in reaction mixtures (12 µl) containing 25 mM Tris-HCl (pH 7.5), 40 mM sodium acetate, 10 mM magnesium acetate, 20 mM 2-mercaptoethnol, 0.25 mg/ml BSA, 0.5 mM ATP-γ-S, and various labeled substrates (20 fmole for DNA binding and 10 fmole for helicase assays). In DNA binding assays, complexes were separated on a 5% native polyacrylamide gel after incubation at 30°C for 30 min. In DNA helicase assay, after incubation of the above reaction mixtures at 30°C for 30 min, ATP (final 10 mM) was added and incubation was continued at 37°C for 30 min. After termination of the reaction by the addition of stop buffer (final 20 mM EDTA and 0.1% sodium dodecyl sulfate [SDS]), the samples were separated on a non-denaturing polyacrylamide gel in 1x TBE buffer.

### ATPase Assays

ATPase activities were examined in reaction mixtures (12 µl) containing 50 mM Tris-HCl (pH 7.5), 10 mM magnesium acetate, 20 mM 2-mercaptoethnol, 0.5 mg/ml BSA, 1 mM ATP (with 2 µCi of [γ-^32^P]ATP), and 87 mer oligonucleotide (20 pmole). After incubation at 30°C for 1 hr, aliquots were spotted onto a polyethyleneimine-cellulose thin layer plate, and ATP and Pi were separated by chromatography in 1 M formic acid and 0.5 M LiCl. The radioactivity on the plate was detected by using a Bio-Image analyzer (BAS 2500; Fuji).

### Immuno-precipitation Analyses

The primase (His-p58/p48 complex) was mixed with purified Mcm2∼7 containing a Flag-tag in Mcm5 in the presence or absence of GINS complexes, and immuno-precipitation was performed using anti-Flag M2 antibody agarose beads (Sigma). The pre-washed anti-Flag antibody beads were mixed with the proteins with or without an oligonucleotide in a buffer containing 50 mM Tris-HCl (pH 7.5), 100 mM sodium acetate, 5 mM magnesium acetate, 1 mM ATP, 10% glycerol, and 0.01% Triton-X-100. As necessary, DNA digestion with 1 U of DNase I in above buffer was performed. After a 2-hr incubation at 4°C, beads were washed three times with the same buffer and bound proteins were eluted with 0.1 M glycine (pH 2.8) twice, or the beads were directly boiled after addition of sample buffer. The samples were analyzed by SDS-polyacrylamide gel electrophoresis (PAGE) followed by silver staining, SPYRO staining or western-blotting with anti-penta-His antibody, anti-p48 antibody or anti-GINS antibody.

### Immuno-precipitation Experiments on Insect Cells Co-infected with Baculoviruses

Sf9 cells were seeded at 5×10^6^ in 10 cm dishes, infected with appropriate baculoviruses and incubated at 27°C. At 72 hrs after infection cells were harvested, centrifuged at 1000-g for 5 min and cell pellets were re-suspended in the lysis buffer (40 mM HEPES-NaOH, pH 7.5, 100 mM sodium acetate, 1 mM DTT, 10 mM magnesium acetate, 1 mM EDTA, 0.1% NP-40, protease and phosphatase inhibitors tablets [Roche]). Cells were lysed using a Soniprep 150 (Sanyo) at 10 amplitude microns, four times for 10 sec each. One tenth of the supernatant was mixed with 10 µl of M2 Flag-Agarose affinity gel. Samples were washed five times with the lysis buffer and eluted with the lysis buffer containing 0.3 mg/ml of Flag peptide. One fifth of the immuno-precipitated samples were loaded on SDS-PAGE, blotted and detected with anti-Flag or other antibodies indicated.

### Glycerol Gradient Fractionation

Purified Mcm4/6/7 complex alone or the mixtures of DNA polymerase α-primase and Mcm4/6/7 complexes were loaded onto a 15–30% glycerol gradient in 20 mM Tris-HCl (pH 7.5), 150 mM NaCl, 0.5 mM EDTA, 1 mM DTT, 0.01% Triton X-100, and 0.1 mM phenylmethylsulfonyl fluoride (PMSF). Since protein concentration was low, heat-denatured α-Casein (10 µg; from Sigma) was added for stabilization. After centrifugation at 36,000 rpm in a TLS-55 rotor for 16 hrs at 4°C, 15 fractions were collected from the top of the gradient. Purified Mcm4/6/7 or Mcm2∼7 complex and human DNA primase, singly or in combination, were fractionated in a 15–35% glycerol gradient in 20 mM Tris-HCl (pH 7.5), 100 mM NaCl, 1 mM ATP, 5 mM magnesium acetate, 0.5 mM EDTA, 1 mM DTT, 0.01% Triton X-100, and 0.1 mM PMSF at 36,000 rpm in a TLS-55 rotor for 18 hrs at 4°C in the presence of heat-denaturedα-Casein (10 µg).

### DNA-binding Analyses using Biotin-labeled ssDNA

The primase and Mcm2∼7 or Mcm4/6/7 complexes were mixed with 50 pmole of biotin-labeled 37mer-dT_40_ oligonucleotide in a buffer (20 µl) containing 25 mM Tris-HCl (pH 7.5), 100 mM sodium acetate, 10 mM magnesium acetate, 0.25 mg/ml Casein, 1 mM DTT, 0.01% Triton-X, and 0.5 mM ATP-γ-S or 1 mM ATP. After incubation at 30°C for 30 min, streptavidin-coated M-280 magnetic beads (Dynal Biotech ASA) were added to the mixture and incubated for 30 min at 4°C. After the removal of the supernatant (unbound), the beads were washed four times in a buffer (400 µl) containing 20 mM Tris-HCl (pH 7.5), 100 mM sodium acetate, 10 mM magnesium acetate, 1 mM DTT, 0.01% Triton X-100, and 10% glycerol, re-suspended in SDS sample buffer (bound), and were analyzed on 4–20% SDS-PAGE, followed by silver staining.

## Supporting Information

Figure S1 **Purified Mcm4/6/7 and Mcm2∼7 complexes.** Purified Mcm2/4/6/7 (lane 1, 700 ng), Mcm2∼7 (lane 2, 300 ng; lane 3, 600 ng) and Mcm4/6/7 (lane 4, 100 ng) complexes, analyzed on a 5% native gel, migrated as 400 kDa, 600 kDa and 550 kDa complexes, respectively. The proteins were detected with silver staining.
(TIF)

Figure S2 **Direct interaction between Mcm helicase and GINS complex and identification of the subunits of Mcm interacting with primase.** (A) The purified Mcm2∼7 complex (1 μg) was mixed with the GINS complex (1 μg) and immuno-precipitation was performed using anti-Flag M2 antibody beads. The bound proteins were eluted with 0.1 M glycine (pH 2.8). The eluted samples (B) and 1/10 (for SPYRO staining) or 1/20 (for western analyses) of unbound fractions (U) were analyzed by SYPRO staining or immuno-blotting with antibodies indicated. Samples were run on 4–20% gradient gel. (B) Mcm subunits 2, 5, or 6 were not co-immuno-precipitated with the human DNA primase (related to Figure 1G).
(TIF)

Figure S3 **Complex formation of Mcm4/6/7 or Mcm2∼7 and primase analyzed in a native gel.** The primase complex (p48/p58; 200 ng (A) or 275 ng (B)), Mcm4/6/7 complex (200 ng), Mcm2∼7 complex (400 ng) and a mixture of primase plus the Mcm complex were incubated in a reaction mixture containing 25 mM Tris-HCl (pH 7.5), 5 mM magnesium acetate, 20 mM 2-mercaptoethnol, 0.01% Triton X-100, and 1mM ATP at 30°C for 15 min, and the samples were analyzed on 5% native-PAGE at 4°C, followed by silver staining. Thyroglobulin (669 kDa), ferritin (440 kDa), catalase (232 kDa), and lactate dehydrogenase (140 kDa) (GE Healthcare) were used as protein molecular weight markers.
(TIF)

Figure S4 **Effect of primase on DNA-binding activity of Mcm4/6/7 (related to** **Figure 3C** **).** (A) DNA-binding assays on oligonucleotide DNA (37mer-dT_50_) were repeated. A constant amount of Mcm4/6/7 (50 ng) or various amounts of Mcm4/6/7 (25 ng, 50 ng and 100 ng), and a constant amount of p48/p58 (50 ng) or various amounts of the p48/p58 primase (25 ng, 50 ng and 100 ng) were added. The samples were run in 5% native gel containing 5% glycerol, 0.5x TBE, and acrylamide: bis (37.5:1). The reproducible result was observed. (B) The same combinations of proteins as in lanes 1–8 of Figure 3C were incubated with a 132mer oligonucleotide DNA. The samples were run in 5% native gel containing 1x TBE and acrylamide: bis (32.3:1).
(TIF)

Figure S5 **Effect of primase proteins on Mcm helicase activity.** DNA helicase activity was examined with a constant amount of Mcm4/6/7 (40 ng) and primase proteins (50 ng, 100 ng, and 200 ng). DNA helicase assays were conducted using the partial hetero-duplex substrate (15 fmoles) in reaction mixture. After incubation at 37°C for 1 hr, the reactions were terminated directly by addition of EDTA (20 mM) and SDS (0.1%) (lanes 1–5) or by the addition of 4 µg/ml proteinase K and 0.1% SDS (37°C, 15 min) followed by the addition of EDTA (20 mM) (lanes 7–11). The samples were then separated by electrophoresis on a non-denaturing polyacrylamide gel in 1x TBE. 25-fold cold competitor oligonucleotide DNA (37mer-dT_40_) was present in all the reactions.
(TIF)

Figure S6 **Single-stranded DNA binding activities of primase and Mcm2∼7 complex (related to** **Figure 6A** **).** The unbound supernatant fractions from Figure 6A were analyzed on 4–20% SDS-PAGE, followed by silver staining.
(TIF)
