# Supplementary figures and images for: The Mini-Chromosome Maintenance (Mcm) Complexes Interact with DNA Polymerase α-Primase and Stimulate Its Ability to Synthesize RNA Primers
Source: PLoS One. 2013 Aug 20;8(8):e72408. doi: 10.1371/journal.pone.0072408 (PMC3748026; doi:10.1371/journal.pone.0072408)

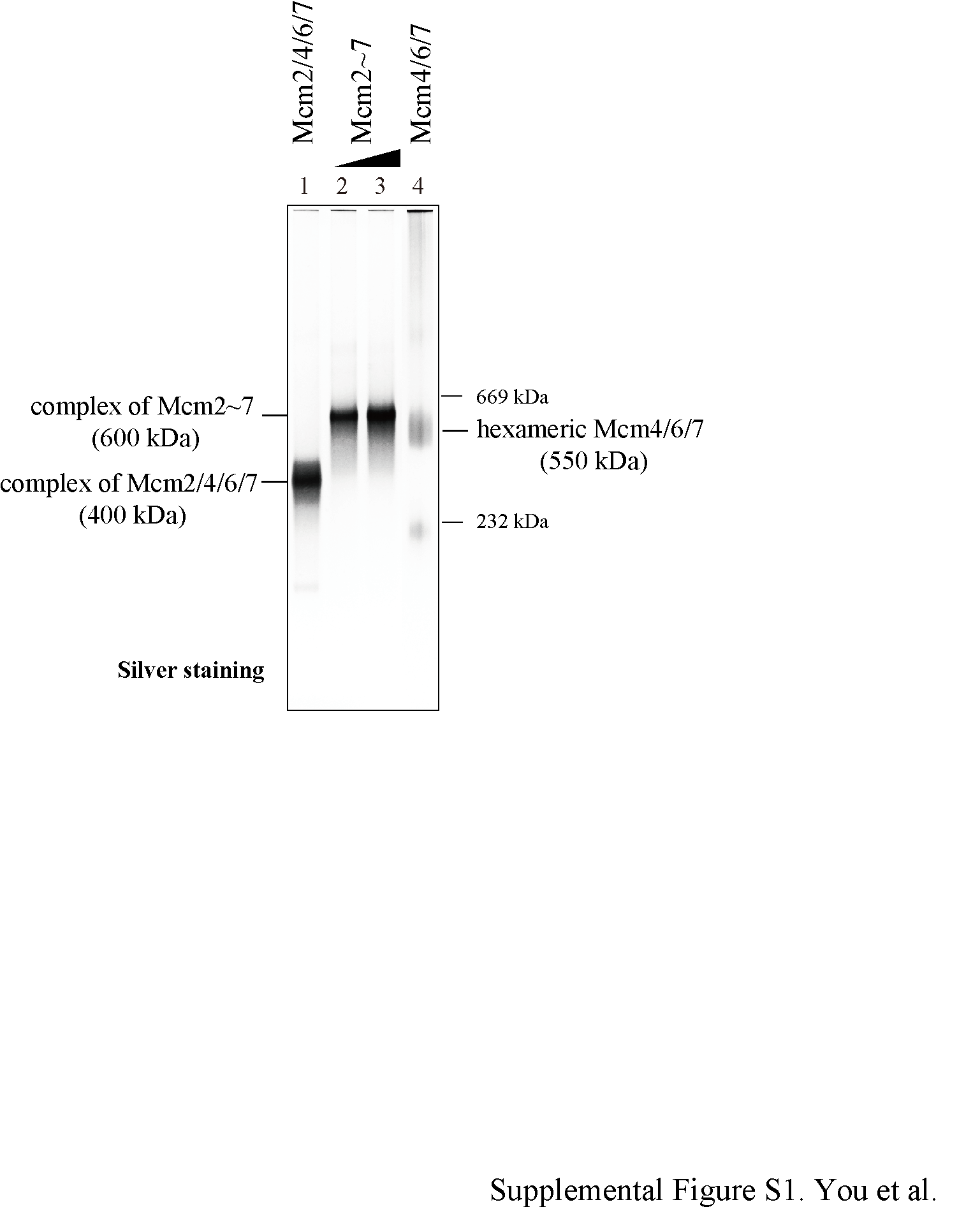

Supplement: Figure S1 — Purified Mcm4/6/7 and Mcm2∼7 complexes. Purified Mcm2/4/6/7 (lane 1, 700 ng), Mcm2∼7 (lane 2, 300 ng; lane 3, 600 ng) and Mcm4/6/7 (lane 4, 100 ng) complexes, analyzed on a 5% native gel, migrated as 400 kDa, 600 kDa and 550 kDa complexes, respectively. The proteins were detected with silver staining. (TIF) [file pone.0072408.s001.tif]

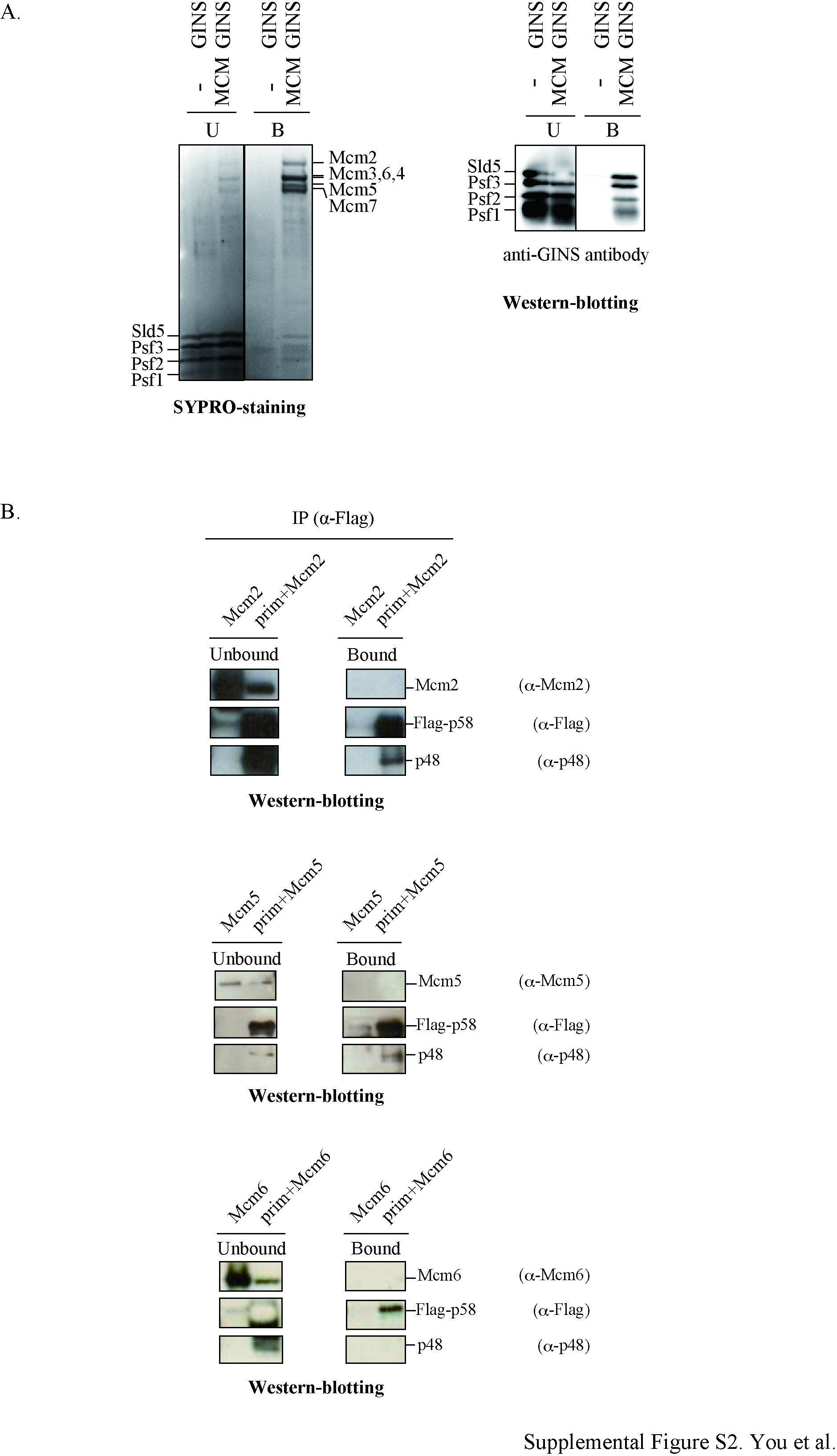

Supplement: Figure S2 — Direct interaction between Mcm helicase and GINS complex and identification of the subunits of Mcm interacting with primase. (A) The purified Mcm2∼7 complex (1 μg) was mixed with the GINS complex (1 μg) and immuno-precipitation was performed using anti-Flag M2 antibody beads. The bound proteins were eluted with 0.1 M glycine (pH 2.8). The eluted samples (B) and 1/10 (for SPYRO staining) or 1/20 (for western analyses) of unbound fractions (U) were analyzed by SYPRO staining or immuno-blotting with antibodies indicated. Samples were run on 4–20% gradient gel. (B) Mcm subunits 2, 5, or 6 were not co-immuno-precipitated with the human DNA primase (related to Figure 1G). (TIF) [file pone.0072408.s002.tif]

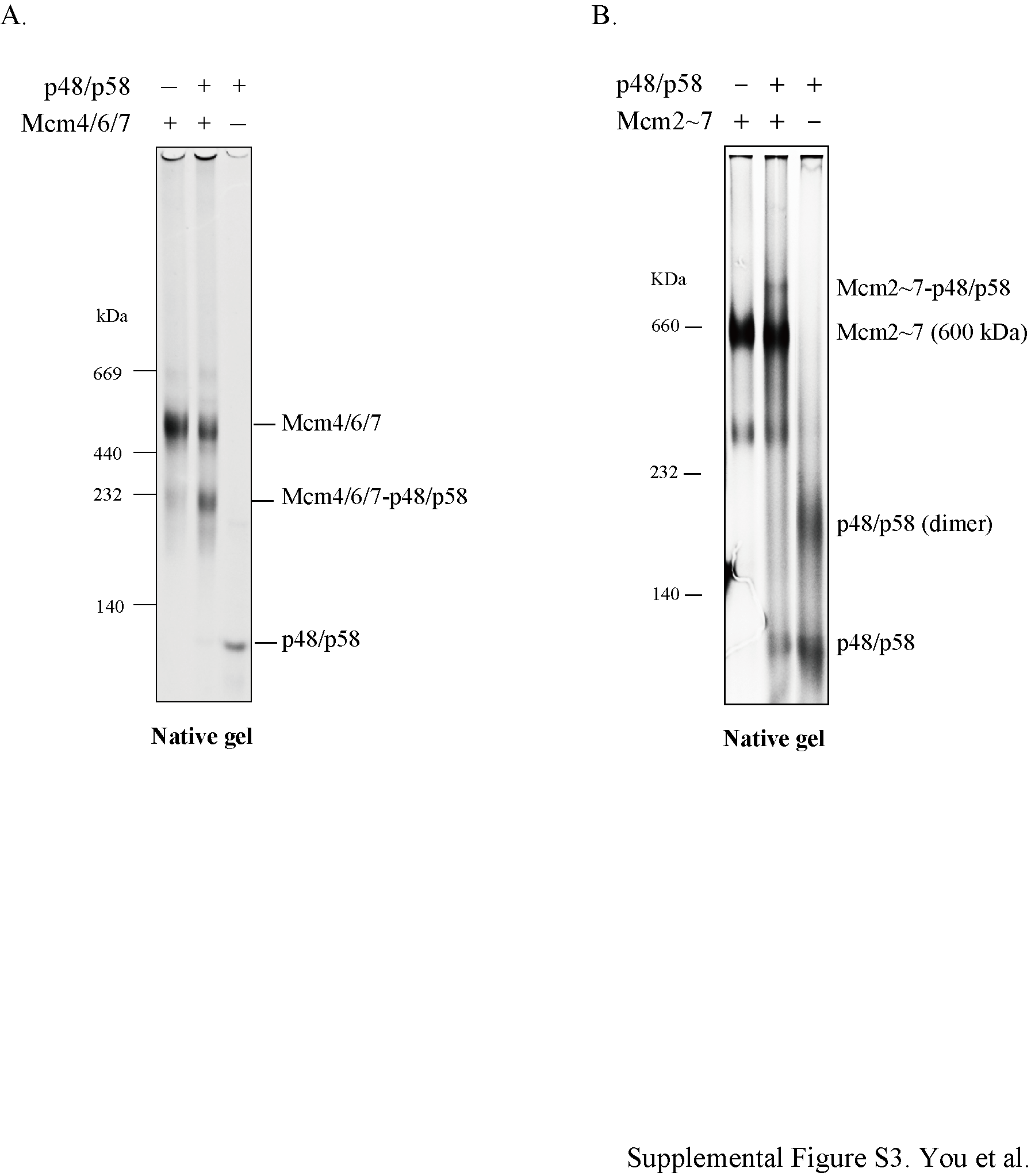

Supplement: Figure S3 — Complex formation of Mcm4/6/7 or Mcm2∼7 and primase analyzed in a native gel. The primase complex (p48/p58; 200 ng (A) or 275 ng (B)), Mcm4/6/7 complex (200 ng), Mcm2∼7 complex (400 ng) and a mixture of primase plus the Mcm complex were incubated in a reaction mixture containing 25 mM Tris-HCl (pH 7.5), 5 mM magnesium acetate, 20 mM 2-mercaptoethnol, 0.01% Triton X-100, and 1mM ATP at 30°C for 15 min, and the samples were analyzed on 5% native-PAGE at 4°C, followed by silver staining. Thyroglobulin (669 kDa), ferritin (440 kDa), catalase (232 kDa), and lactate dehydrogenase (140 kDa) (GE Healthcare) were used as protein molecular weight markers. (TIF) [file pone.0072408.s003.tif]

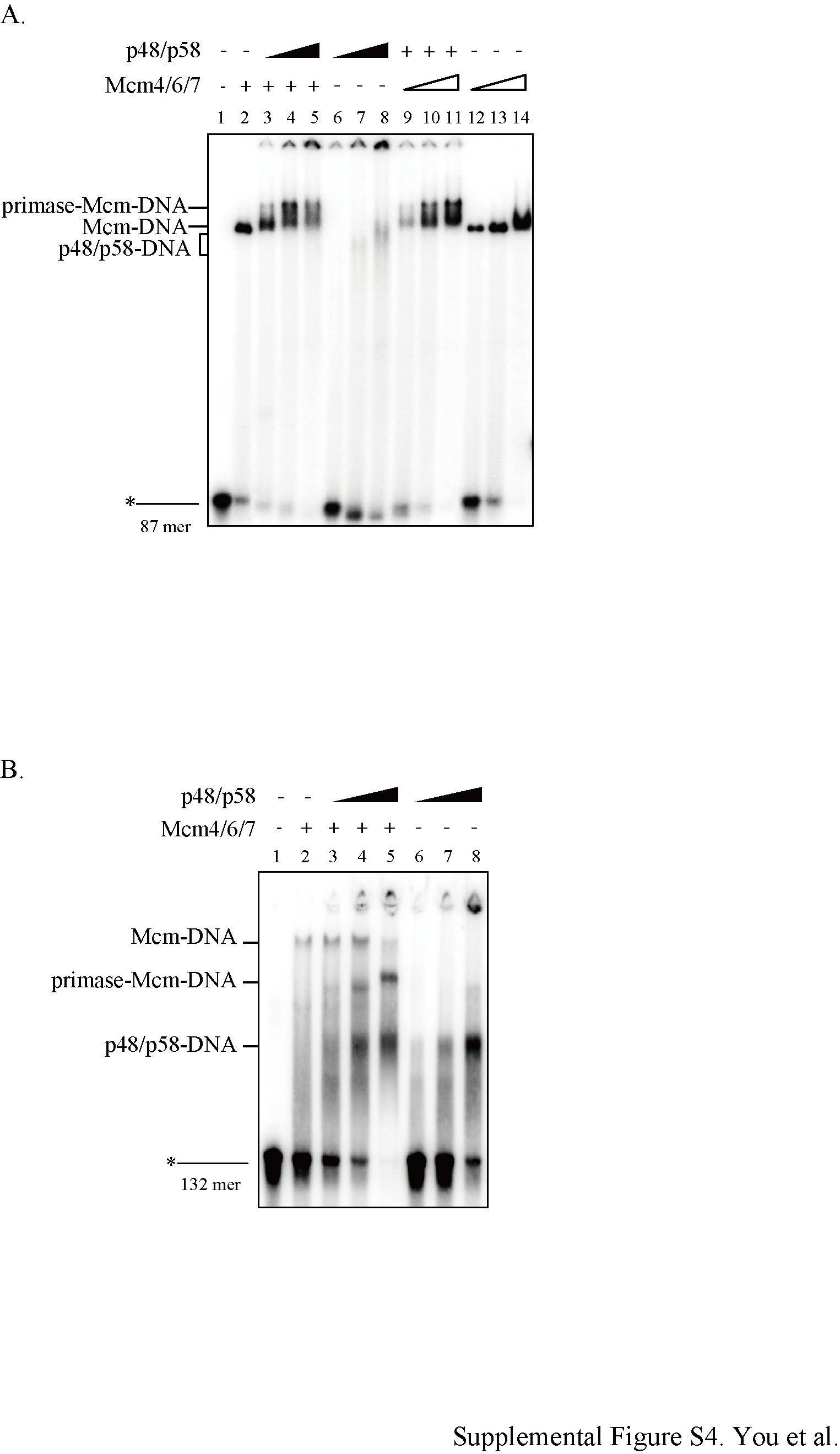

Supplement: Figure S4 — Effect of primase on DNA-binding activity of Mcm4/6/7 (related to Figure 3C ). (A) DNA-binding assays on oligonucleotide DNA (37mer-dT50) were repeated. A constant amount of Mcm4/6/7 (50 ng) or various amounts of Mcm4/6/7 (25 ng, 50 ng and 100 ng), and a constant amount of p48/p58 (50 ng) or various amounts of the p48/p58 primase (25 ng, 50 ng and 100 ng) were added. The samples were run in 5% native gel containing 5% glycerol, 0.5x TBE, and acrylamide: bis (37.5:1). The reproducible result was observed. (B) The same combinations of proteins as in lanes 1–8 of Figure 3C were incubated with a 132mer oligonucleotide DNA. The samples were run in 5% native gel containing 1x TBE and acrylamide: bis (32.3:1). (TIF) [file pone.0072408.s004.tif]

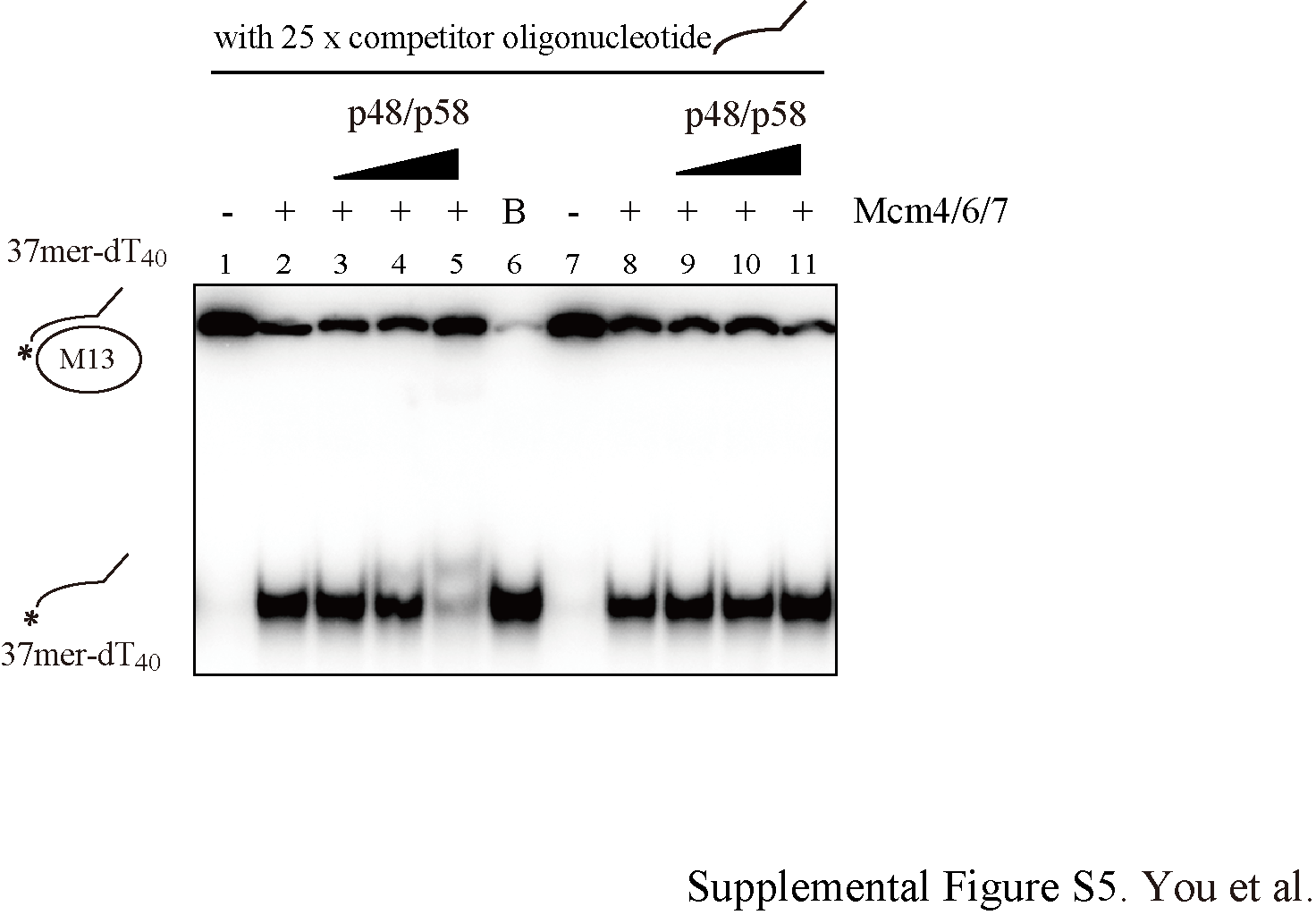

Supplement: Figure S5 — Effect of primase proteins on Mcm helicase activity. DNA helicase activity was examined with a constant amount of Mcm4/6/7 (40 ng) and primase proteins (50 ng, 100 ng, and 200 ng). DNA helicase assays were conducted using the partial hetero-duplex substrate (15 fmoles) in reaction mixture. After incubation at 37°C for 1 hr, the reactions were terminated directly by addition of EDTA (20 mM) and SDS (0.1%) (lanes 1–5) or by the addition of 4 µg/ml proteinase K and 0.1% SDS (37°C, 15 min) followed by the addition of EDTA (20 mM) (lanes 7–11). The samples were then separated by electrophoresis on a non-denaturing polyacrylamide gel in 1x TBE. 25-fold cold competitor oligonucleotide DNA (37mer-dT40) was present in all the reactions. (TIF) [file pone.0072408.s005.tif]

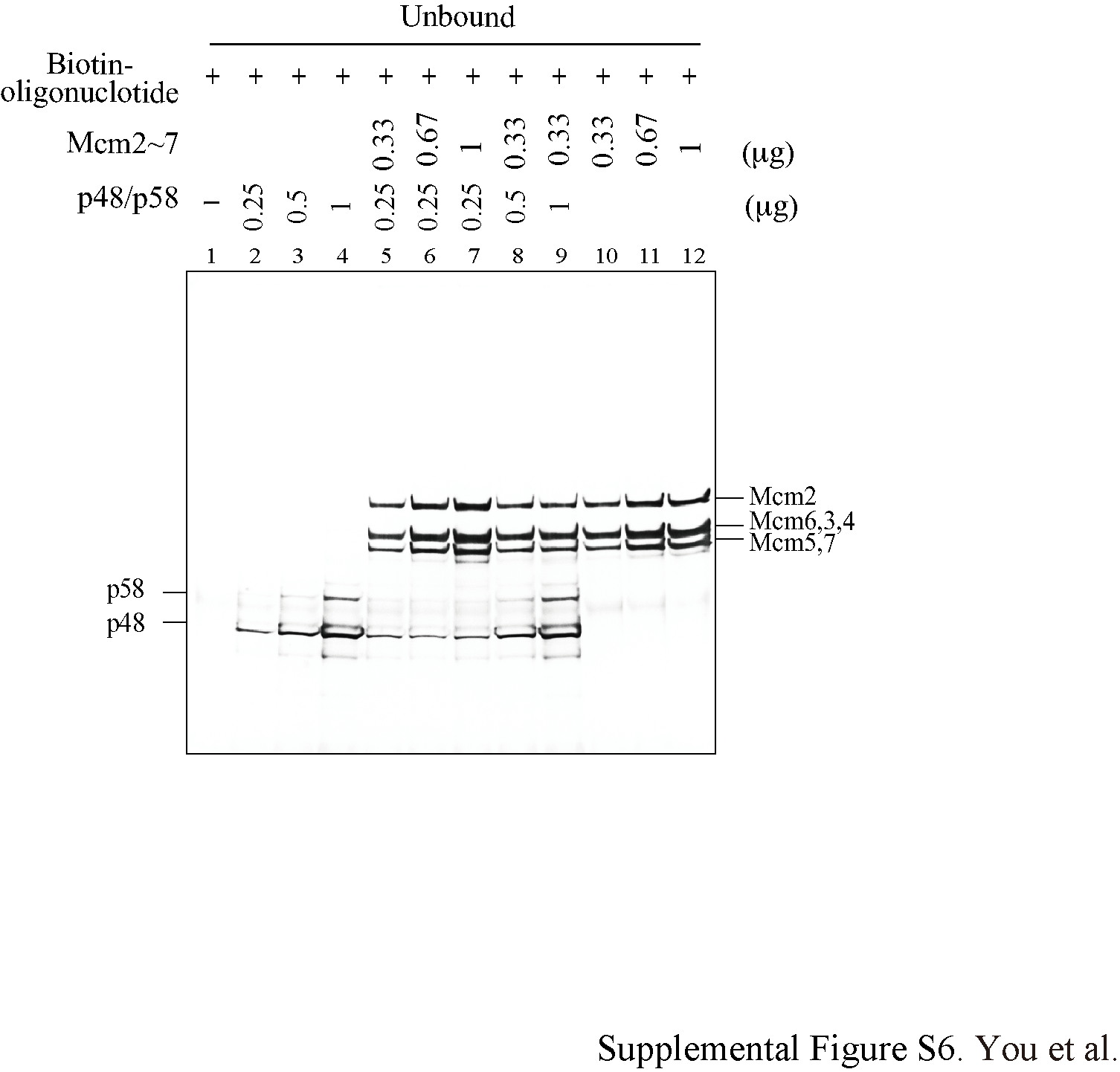

Supplement: Figure S6 — Single-stranded DNA binding activities of primase and Mcm2∼7 complex (related to Figure 6A ). The unbound supernatant fractions from Figure 6A were analyzed on 4–20% SDS-PAGE, followed by silver staining. (TIF) [file pone.0072408.s006.tif]
